# Supplementary material for: Development of a computer-aided design software for the quantitative evaluation of aesthetic damage
Source: PLoS One. 2019 Dec 18;14(12):e0226322. doi: 10.1371/journal.pone.0226322 (PMC6919621; doi:10.1371/journal.pone.0226322)
Supplement: S1 Code site — 1. (ZIP) [file pone.0226322.s001.zip › [EESC_jr][Projeto]Relatorio_07_21/reports/templates_zip/formwizard-master.zip!/formwizard-master/examples/example_19_summary_page.html]

xml version="1.0" encoding="UTF-8"?


JQuery Form Wizard


### Example of a branching wizard with common summary page

- Branching wizard with five steps.
- Branching is decided depending on which country is selected.
- The content (input and labels) of the steps are wrapped with div:s. The div:s have a class="input" set.
- When the summary page is shown, the divs with class="input" are shown in the div with id="summaryContainer".
- When moving back from the summary page, the div:s with class="input" are added back into their normal step.

---


Common first step - Name and Country  

First name  
  
Surname  
  
Country  


Sweden
Finland

Swedish last step - Information  

Social Security Number  


 -
  
Phone number  
 -
 -
  
\*Email  
  
User name  
  
Password  
  
Retype password

Finnish Step 2 - Personal information  

Social Security Number  


 -
  
Phone number  
 -
 -
  
\*Email

Finnish last step - Username  

User name  
  
Password  
  
Retype password

Summary page  

Please verify your information below.


---
